# *Bacillales* isolate genomes from the macroalga *Sargassum ilicifolium* found in Singaporean coastal seawater

**DOI:** 10.1128/mra.00329-25

**Published:** 2025-07-31

**Authors:** Joao Paulo Andre Pereyra, Jessica Alison Taylor, Su Xuan Gan, Prasha Maithani, Rebecca Josephine Case

**Affiliations:** 1 Singapore Centre for Environmental Life Sciences Engineering (SCELSE), Nanyang Technological University (NTU), Nanyang, Singapore; 2 School of Biological Sciences, Nanyang Technological University (NTU), Nanyang, Singapore; Indiana University Bloomington, Bloomington, Indiana, USA

**Keywords:** *Sargassum*, brown alga, *Bacillales*, natural antimicrobial products, Singapore

## Abstract

Four strains belonging to the order *Bacillales* (SSi122, SSi130, SSi176, and SSi177) were isolated from the ubiquitous seaweed *Sargassum ilicifolium* in Singapore. Genomic analysis of these isolates revealed the presence of secondary metabolite gene clusters with the potential for bactericidal and bacteriostatic activity.

## ANNOUNCEMENT

The genera *Priestia*, *Cytobacillus*, *Bacillus,* and *Lysinibacillus* are all members of the order *Bacillales*, frequently identified in terrestrial habitats, though also inhabiting marine niches, including sediments, and can be host-associated (1). Their ubiquity is attributed to their ability to survive stressful conditions, including high salt concentrations and low water availability (2). They can produce many bioactive molecules, which are implicated in cell differentiation and host defense (3).

Thalli from the macroalga *Sargassum ilicifolium* were collected from the northern side of St. John’s Island, Singapore, stamped onto enrichment agar (2.5% wt/vol BBL Brain Heart Infusion broth, 1.5% wt/vol Instant Ocean Sea Salt, 0.03% wt/vol K_2_TeO_3_, and 1% vol/vol Tween 80), and incubated at 30°C for 24 h (4). Bacterial isolates were differentiated based on phenotype, and individual colonies were subcultured using the same media to ensure purity. For each isolate, single colonies were used to inoculate liquid and solid enrichment medium, grown aerobically for 24 h, harvested, and washed with PBS solution.

DNA was extracted with the DNeasy Blood & Tissue Kit (Qiagen, Hilden, Germany), as per the manufacturer’s instructions for gram-positive bacteria, with the addition of RNase A. DNA libraries were prepared using the TruSeq Nano DNA Library Prep Kit (Illumina, San Diego, CA, USA) and sequenced using an Illumina HiSeq X Ten platform, v2.5, generating 150 bp paired-end reads, at the SCELSE Sequencing Facility, NTU.

Trimmomatic version 0.39 (5) was used for adapter trimming and quality filtering of raw reads. Default parameters were used for all software unless specified. Genomes were assembled (Table 1) using the Shovill pipeline v1.1.0 (https://github.com/tseemann/shovill) that used SPAdes v3.15.5 (6) for assembly. Annotation of the assembled genomes was carried out with PGAP v6.7 (7). A putative pBM200 circularized plasmid (7,447 bp) was found in SSi130 (for Singaporean *S. ilicifolium* isolate) using PlasmidFinder v2.0.1 (8).

**TABLE 1:** Genome features and GenBank accession numbers of the four *Bacillales* isolates

Isolate	Phenotype (colony description)^*a*^	Genome size (bp)	No. of CDS^*b*^	No. of rRNAs	No. of tRNAs	G + C content (%)	No. of contigs	*N*_50_ (bp)	*L*_50_	Assembly accession no.	SRA accession no.	No. of reads	Average read length (bp)
*Lysinibacillus fusiformis* SSi122	Small-sized, creamy-white, glossy	4,809,670	4,683	20	94	37.3	61	342,097	3	JBMIIA000000000	SRR32726464	8,537,796	151
*Priestia megaterium* SSi130	Small- to medium-sized, yellow-white, glossy	5,523,822	5,697	20	124	37.8	103	496,025	3	JBMIHZ000000000	SRR32726463	6,666,157	151
*Cytobacillus firmus* SSi176	Medium- to large sized, translucent with cream-white centers, smooth and glossy	4,596,251	4,658	18	105	41.7	262	76,877	15	JBMIHY000000000	SRR32727693	7,406,593	151
*Bacillus altitudinis* SSi177	Large-sized, translucent with cream-white centers, smooth and glossy	3,744,508	3,797	9	74	41.3	24	704,353	3	JBMIHX000000000	SRR32727692	7,568,622	151

^*a*^ Description when grown on marine broth (Difco 2216) agar.

^*b*^ CDS, coding DNA sequences.

 Isolates were identified using the Genome Taxonomy Database Toolkit (GTDB-Tk) v1.7.0 (9) using topology and average nucleotide identity (ANI). Results showed SSi122 had the greatest similarity to *Lysinibacillus fusiformis* (GCF003049525), SSi130 to *Priestia megaterium* (GCF009497655), SSi176 to *Cytobacillus firmus* (GCF001591465), and SSi177 with *Bacillus altitudinis* (GCF000691145). Phylogenetic relatedness between each isolate genome and publicly available reference genomes was performed using the Orthologous Average Nucleotide Identity Tool v0.93.1 (10) and the Genome-To-Genome Distance Calculator v3.0 (11), to calculate the ANI and digital DNA-DNA hybridization (dDDH) scores, respectively. All genomes were <95% ANI and <70% dDDH to each other, and >95% ANI and >70% dDDH to their GTDB-Tk identified reference genomes.

 Biosynthetic gene cluster analysis using antiSMASH v7.1.0 (12) revealed that the genomes of *P. megaterium* SSi130 and *B. altitudinis* SSi177 encoded several clusters. SSi130 contained genes for the production of the lasso peptide (known antibacterials) paeninodin (13, 14) and the cyclic dipeptide pulcherrimic acid, a bacteriostatic iron chelator (15). SSi177 contained gene clusters for the production of bacilysin (an algaecide against harmful algal bloom species) (16), the cannibalism-mediating sporulation killing factor (17), and the biosurfactant lychenisin, used in microbially enhanced oil recovery (18).

## Data Availability

The SRA data were deposited to NCBI, and the complete genome sequences was deposited to GenBank under accession numbers listed in Table 1.
